# Rest to Promote Learning: A Brain Default Mode Network Perspective

**DOI:** 10.3390/bs14040349

**Published:** 2024-04-22

**Authors:** Wei Luo, Biao Liu, Ying Tang, Jingwen Huang, Ji Wu

**Affiliations:** 1 Department of Applied Psychology, School of Education Sciences, Nanning Normal University, Nanning 530299, China; 040037@nnnu.edu.cn (W.L.); ty1420814367@163.com (Y.T.); 2 Institute of Psychology, Chinese Academy of Sciences, Beijing 100101, China; 3 Guangxi Education Modernization and Quality Monitoring Research Center, Nanning 530001, China; 4 School of Foreign Languages, Nanning Normal University, Nanning 530100, China; liubiao@nnsfdx.wecom.work; 5 Department of Science Research, Guangxi University, Nanning 530004, China; kingwen@gxu.edu.cn

**Keywords:** learning, rest, the default mode network (DMN), neural mechanism

## Abstract

The brain often switches freely between focused attention and divergent thinking, and the Default Mode Network (DMN) is activated during brain rest. Since its discovery, the DMN, together with its function and characteristics, indicates that learning does not stop when the brain “rests”. Therefore, DMN plays an important role in learning. Neural activities such as beta wave rhythm regulation, “subconscious” divergence thinking mode initiation, hippocampal function, and neural replay occur during default mode, all of which explains that “rest” promotes learning. This paper summarized the function and neural mechanism of DMN in learning and proposed that the DMN plays an essential role in learning, which is that it enables rest to promote learning.

## 1. Introduction

Nowadays, the application of cognitive neuroscience results in the field of education has become one of the research focuses [1]. The Default Mode Network (DMN) is a novel and recently valued brain system that participates in internal cognitive patterns [2]. DMN has made people realize the importance of rest in learning, changed the cognition of human brain function and learning, completely overturned the understanding of the working mechanism of the human brain [3], and subverted the understanding of “rest”. Resting state DMN activity may be associated with wisdom performance [4].

To focus well, the individual should learn to take breaks and “not to get focused”. If the learning time exceeds a certain period, the effect will gradually decrease, and if the learning time is extended, it will be difficult to have an effect [5]. Brain science research results show that one cannot learn efficiently without a good rest [6], as rest promotes learning which continues when people rest their brain. A scientific brain break will make learning easier and more enjoyable. The attempt to double down and force concentration often leads to poorer performance. Learning from dawn to dusk on a tight schedule can leave a person physically and mentally exhausted, resulting in reduced efficiency. Similarly, trying to study without any distractions every second may also lead to unmet expectations. It’s essential to mobilize attention resources effectively, but it’s equally important to take breaks and relax moderately to maintain high productivity. Mobilizing the resources of attention all the time requires the continuous working of the related brain regions, which results in brain “strike”. Meanwhile, the brain is unable to switch to other functional regions for information integration and sorting. People who devote the longest time to studying are not necessarily high in efficiency, and the efficient performers all have one thing in common; that is, they manage their study time well, take breaks, and complete necessary tasks while maintaining energy [7]. This paper concentrates on the aspect of learning and explains the neural mechanism of rest which promotes learning from the perspective of DMN.

DMN has been a hot topic in neuroscience for more than two decades [3]. In mental health research, the association between DMN and various mental diseases has been widely explored, but most of them focus on the clinical application of mental diseases [8,9,10]. For example, the DMN activity of patients with depression may be abnormally enhanced, which leads to excessive self-reflection and negative thinking [10]. At present, the potential role of the DMN in learning process has drawn more and more attention from researchers, although this research direction is relatively new [11,12,13]. From the perspective of the DMN, brain activity in the non-task state often conceptualized as “rest” may facilitate cognitive processes, especially learning. This paper aims to propose a novel learning insight to dialectically understand the relationship between the “resting” and “learning” states of the brain by recognizing the positive role of the DMN in non-task-driven cognitive functions. It is possible that DMN activity is not merely a background for mind wandering, but is involved in the consolidation of memory, the formation of innovative thoughts, and the internalization process of knowledge. Exploring this area may help to promote the application of neuroscience research results in educational practice, while providing a scientific basis for educational activities. For example, learning protocols that incorporate appropriate rest periods may be more effective in promoting the long-term retention of information and the overall cognitive development of students. In the long run, these studies may have a profound impact on the development of educational strategies, the optimization of learning methods, and the improvement of learning efficiency.

## 2. The Role of DMN in Learning

**Figure 1:**
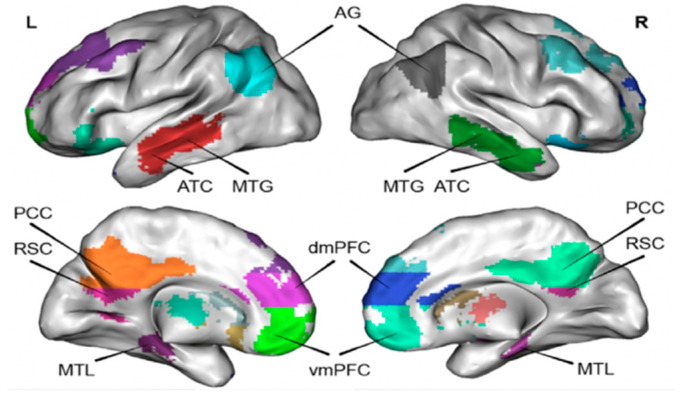
Brain Default Mode Network. Adapted from Menon [3]. Note: angular gyrus (AG), anterior temporal cortex (ATC), middle temporal gyrus (MTG) retrosplenic cortex (RSC), posterior cingulate cortex (PCC), medial temporal lobe (MTL), dorsalmedial prefrontal cortex (dmPFC), and ventromedial prefrontal cortex (vmPFC).

A number of studies have found that parts of the brain’s association cortex are inhibited during tasks that require external attention, but they become active during the process of memorizing, imagining the future, and making social inferences [2,12]. It is generally believed that the resting state refers to the state of being awake, eyes closed, or no specific cognitive task. Researchers discovered that a fixed region of the brain is active in the resting state, there is no difference between the metabolic rate of these regions and the task execution state, and there is continuous and active neural activity [3,14]. Additionally, frontal lobe activity reaches a high level at rest and still carries out brain work, and the frontal lobe activity pattern corresponds to non-directional, spontaneous, and conscious mental states [2]. Raichle et al. [15] proposed the hypothesis of resting state activity based on the fact that certain brain regions present strong signals and perform strong and regular activities in the waking resting state of the human brain. In 2001, Raichle et al. [16] formally proposed the concept of the brain DMN, and it was not until 2016 that Zhu et al. [17] used functional magnetic resonance imaging (fMRI) technology to verify the existence of the DMN for the first time. Shulman et al. [18] found that the brain DMN is almost inactive or shows negative activation (deactivation) under the condition of positron emission tomography (PET) and fMRI cognitive task experimental conditions. The activity level of this brain region was higher in the resting state than in the task condition [18]. The DMN is a very important and continuously running organizational structure network of the brain in the resting state, which plays a very important role in the monitoring of the internal and external environment, emotional control, and emotional memory extraction [19]. The DMN is composed of discrete, bilaterally symmetrical brain regions, including the posterior cingulate cortex/Precuneus (PCC/Precuneus), dorsalmedial prefrontal cortex (dmPFC), ventromedial prefrontal cortex (vmPFC), angular gyrus (AG), retrosplenic cortex (RSC), anterior temporal cortex (ATC), middle temporal gyrus (MTG), hippocampus/medial temporal lobe (hippocampus/MTL), and other structures [18,19]. The brain DMN structure and location are shown in Figure 1.

The discovery of the DMN indicates that the DMN brain region is activated at rest, but learning does not stop at that moment. When individuals do not pay attention to the external environment, the DMN prioritizes being active [2]. During task-free waking, resting state, or rest, the DMN brain region changes from being silent to active, and there is spontaneous neural activity, the level of which significantly surpasses other brain regions [2]. At this time, the DMN does not rest but performs a specific brain function, namely learning related activities.

## 3. The DMN’s Functions in Learning

**Figure 2:**
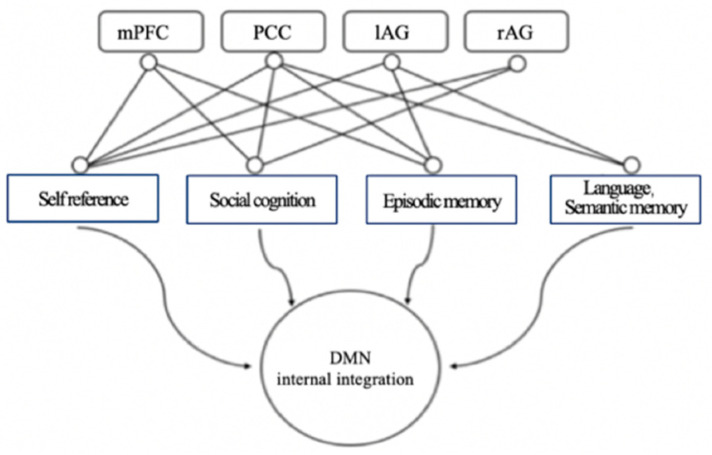
DMN functional map. Adapted from Menon [3]. Note: left angular gyrus (lAG), right angular gyrus (rAG), and medial prefrontal cortex (mPFC).

The DMN’s functions are related to learning. It involves a set of brain regions that are functionally connected to each other [20] and exhibit nonactivation or negative activation in most external tasks [18]. The DMN is activated during internal psychological activities such as self-reference, episodic memory retrieval, moral judgment, and future imagination [12,15,17,21]. The DMN, which is the neural basis of the self, collects memories of events and facts, gathers attributes and descriptions about the self, and reflects on the emotional state. The DMN is also involved in episodic memory [22] and absentmindedness [23]. The DMN has functions such as monitoring the internal and external environment of the brain, emotional processing, introspecting, maintaining thinking and cognition, and recalling thoughts [24]. The DMN plays a role in looking back at the past and looking forward to the future, forming episodic memory to deepen the understanding of an event [12]. (See Figure 2 for DMN functional map) Medial prefrontal cortex, bilateral angular gyrus, and cingulate/anterior cuneus are, respectively, related to social cognition, self-reference, episodic memory, language, and semantic memory [3].

**Figure 3:**
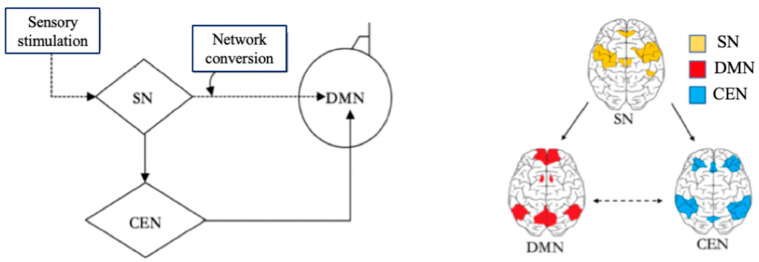
Triple Network model. Adapted from Menon [26]. Note: Salience Network (SN) and Central Executive Network (CEN).

With the help of the DMN, the Central Executive Network (CEN), and the Salience Network (SN), the brain can switch freely between focused attention and divergent thinking, which helps the brain to get efficient rest. When the brain focuses attention, it is in the CEN state [24]. When the brain does not focus attention, it enters the default mode state. The CEN is a specific brain region that is activated during the completion of a task, and it is responsible for preventing interruptions and limiting irrelevant stimuli while people are engaged in work. When the CEN is activated, people will quickly focus their attention on solving the immediate task, which helps to complete the task more attentively and efficiently. The brain turns on the DMN immediately after a task is completed or stopped. There is a certain anticorrelation between the activities of the DMN and the CEN [18]. The CEN is negatively correlated with DMN, as one network is activated while the other will be dormant. After the brain has been in the CEN for a long time, only when the brain is properly relaxed and the DMN in the brain is fully invoked can the brain think more flexibly to generate more different inspirations. The Salience Network (SN) is responsible for regulating the brain switching between the CEN and the DMN [18]. The SN is related to self-awareness, involved in detecting and filtering sensory stimuli, and mobilizing relevant functional networks [25]. The SN prompts the brain to activate the CEN and DMN according to the needs of the external or internal information processing [26]. In the brain, the SN is responsible for regulating the mechanism of the switch between the DMN and the CEN [26], which also indicates that the brain itself needs to constantly switch between work and rest, so as to alternate work with rest. The relationships among the SN, CEN, and DMN are shown in Figure 3 in a Triple Network model.

## 4. Learning-Related DMN Characteristics

The learning requirement leads to the high energy consumption of the DMN. Individuals have different learning tasks in different semesters, and the DMN function matches them accordingly. The DMN also adjusts to different activation states with different cognitive difficulty. Mental illness can lead to DMN dysfunction and disorder, which results in various learning problems. It is also proved from the characteristics of the brain that the DMN is closely related to learning.

### 4.1. DMN’s High Energy Consumption to Meet the Needs of Learning

The energy consumption of the brain in the task state is only about 5% higher than that in the resting state [27]. In the resting state, multiple brain regions of the DMN are always busy, and the energy consumption accounts for 60% to 80% of the total energy consumption of the brain, showing high metabolism in the internal cognitive process [24]. The maximum energy consumption of the brain in the resting state comes from the DMN [28]. Magnetic resonance imaging (MRI) studies have found that the DMN activity that enjoys internal activity consistency is significantly higher than other brain regions, which indicates that DMN has higher metabolism under resting conditions [29,30,31,32]. In the DMN state, the relevant brain regions which are activated run at high speed, and the learning activities are still in full motion, with large energy demand and high metabolism. Compared with other brain networks, the DMN has higher energy consumption to meet the needs of the learning activities in the process of cognitive integration, memorizing, emotional processing, introspection, and so on.

### 4.2. Automatic Adjustment of DMN Activation in Accordance with the Difficulty of Learning Tasks

Specific target tasks can have an effect on the DMN, which is related to the degree of the cognition required by the target task [33]. When participating in simple manual and visual tasks, such as motor, sensory, and perceptual tasks that do not require cognitive participation, the activity of the DMN is almost not affected. The greater the cognitive difficulty is, the more silent the DMN remains, and there is a negative correlation between them [33]. Task load is positively correlated with the negative activation of the DMN [33]. The more difficult the external cognitive task is, the more attention is required, and the CEN operates at a high speed, during which time the DMN is more inhibited. At rest, the DMN needs more time to organize, further study, and digest previous cognitive content.

### 4.3. DMN’s “Inverted U” Shape with Age: The Close Connection with Learning Activities at Different Ages

The DMN is age-related [34], and its functional development changes with age, from being imperfect to perfect, degenerates, and then disappears [33,35,36,37,38]. The DMN changes with age while it is stable at the same time. It is the organization mode of the spontaneous activity of the brain nerve cells, and is related to brain functions such as learning, memory, and cognition [38]. Fair et al. [36] found that the DMN of children around 7 to 9 years old, compared with adults, is not mature, and the functional connection strength between regions in the DMN is not as strong as that of adults [39]. Supekar et al. [33] found that the activity of the medial prefrontal cortex (mPFC) was weak and the functional connectivity strength from PCC to mPFC was also significantly weaker in children; the gray matter volume of the PCC and mPFC regions was larger than that of adults, while the white matter volume was smaller; and the anisotropy of the nerve fibers was greater in adults than that in children. Thomason et al. [39] found that compared with adults, the DMN in children is not mature and gradually develops to the normal adult mode. The DMN in the brain of young children develops from a weakly connected to a gradually strengthened connection, and the network shows the most stable state in adulthood. Marsh et al. [40] used the Stroop paradigm to find that the task-evoked DMN negative activation was positively correlated with age, that is, the older the age, the greater the task-evoked negative activation. The functional connectivity of the anterior and posterior parts of the DMN decreases with age [41,42]. Younger people have more intact DMN components than older people [43], and different ages will lead to different activated regions in the DMN [44]. People in childhood and old age study and work less, while those in adulthood are occupied with work, tasks, and learning, which are all reflected in the development of the DMN, so it shows an “inverted U-shaped” development, which is in line with the principle of the evolution theory.

### 4.4. DMN, Mental Illness and Learning: Mental Illness and Alteration in DMN Affect Each Other and Ultimately Affect Learning

There is a strong correlation between mental disease and alteration in the DMN [45,46,47]. Mental illness and alteration in the DMN affect each other and ultimately affect learning. Alterations in the DMN have been widely observed in neurogenic disorders such as Alzheimer’s disease (AD) [45] and psychiatric disorders [46,47]. Attention deficit hyperactivity disorder (ADHD) is characterized by prolonged and persistent inattention, hyperactivity, and poor impulse control. The functional connectivity between the dorsolateral anterior cingulate and other brain regions in the DMN of patients with ADHD is significantly enhanced [48]. The functional activity of the dorsal anterior cingulate cortex in the DMN decreases in normal children, but this characteristic does not exist in children with ADHD [49]. ADHD may be related to the abnormal frontal–striatum–cerebellum pathway, and the changes in DMN functional connectivity cause related attention deficits and working memory disorders [50]. The locations of the DMN in patients with depression are different, and their activities are abnormal [8,51,52], mainly concentrated in the prefrontal lobe, cingulate gyrus, hippocampus, angular gyrus, and other parts, and the functional connectivity of different regions of the DMN increases or decreases [51,53,54]. The decrease in DMN activation is the main feature of Autism Spectrum Disorder (ASD), and its symptoms occur more in the resting state than in the active moment. The negative activation of the DMN in patients with ASD is abnormal during the Stroop task [55]. The functional connectivity of the medial prefrontal cortex and left angular gyrus of the DMN decreases in patients with ASD, and no abnormality is observed during task activation [56]. The DMN activity of patients with ASD is abnormal [57], and the functional connectivity of the anterior and posterior part of the DMN decreases. The abnormal DMN of ASD may reflect the defects of the theory of mind and self-reference processing ability [56], and the regional consistency of the bilateral PCC/Precuneus in patients with ASD decreases [58]. Mental illness leads to DMN dysfunction and disorder, which affects attention and behavior, thus causing learning activities to be affected. The abnormal activities of the DMN exists in the brain of different types of mental illness, resulting in the failure of its normal functions, which explains why patients with mental diseases have a high incidence of learning problems in reality. Understanding the characteristics of the DMN of mental disease is of great benefit to know the attention characteristics, learning interest, and learning state of individuals. The DMN cannot function properly, which may be the cause of mental illness. The confusion of mental illness is precisely because the balance between the DMN and other brain systems is disrupted [59]. The development and strength of DMN functional connections have a normal trajectory, and there have been research conclusions on the DMN patterns of different ages and genders. If an individual’s DMN representation does not meet their expected characteristics, it may be a pathological change, a sign and judgment marker of mental illness [60].

## 5. Neural Activity Occurring in Default Mode Facilitates Learning

Matthew Berman showed that people with high DMN activation completed tasks at least 10% faster than those with low DMN activation [61]. John Trugakos et al. [62] found that working or studying continuously for 52 min, followed by a 17 min break, can maximize productivity. In the learning process, showing some pictures of baby animals to students for relaxation will make students pay more attention in the subsequent learning and greatly improve the learning efficiency [63]. Taking a holiday or scheduling a break after busy work can reactivate cognitive skills and help improve the ability to solve problems in the future [64]. The human brain is not designed to focus on study and work for a long time every day. In one study, a computer program was developed to remind individuals to take breaks at a fixed time. People who take regular breaks were 13% more accurate at work on average [65]. Proper rest is not a waste of time, as the alternation between work and rest is the key to improving learning efficiency. The brain will consolidate and enhance the memory of the previously learned new knowledge through short breaks, which plays an important role in learning [66]. A number of studies on learning have found that rest promotes learning. Here is a closer look at the neural activity that occurs in the brain during the default mode to explain how rest promotes learning.

### 5.1. The Brain’s Frequent Use of Beta Waves for Rhythmic Regulation during Rest

A study found that the rhythm of beta in brain waves changes during rest in a neural network between the frontal lobes and the parietal lobe, known as the DMN [66]. Enhanced beta waves can regulate individual behavior, help improve attention, enhance memory, and improve problem-solving ability [66]. The brain uses beta waves to consciously switch between different pieces of information, and beta rhythm which serves as a control mechanism determines when information in working memory is read out or cleared, controls when information stored in working memory is expressed, and allows it to influence behavior. In the period of beta rhythm, the millions of neurons in the brain each generate their own signals, and these combined signals produce brain wave oscillations. When the theta wave rhythm increases, the beta wave rhythm decreases and vice versa. The reasonable planning of learning and rest, which helps the brain switch to beta rhythm in time, is necessary to improve learning ability and to strengthen knowledge memory.

### 5.2. The Brain’s Spontaneous Initiation of the “Subconscious” Divergence Mode during Rest

The “conscious” focus mode and the “subconscious” divergence mode are the two modes of the brain which are related to learning [67]. The “conscious” focus mode is equivalent to the activation of the CEN. When focusing on a certain thing or task, the prefrontal cortex automatically transmits signals along the neural pathways, transmitting information to various brain regions related to the task content and connecting them. However, in this mode, the answer is not necessarily found because the answer is not necessarily obtained through the brain regions of conscious attention. Therefore, this raises the need for “subconscious” divergence pattern, which switches into the DMN, taking the brain out of its original working regions and allowing neurons to connect randomly to unrelated regions to find an answer that might solve the problem. In order to make the “subconscious” work, one must meet the condition that the awake “consciousness” is completely shut down to completely forget the original thing, that is, take a rest, and let the brain enter the default mode.

### 5.3. The Hippocampus’ Full Engagement in Information Integration during Rest

At rest, the brain integrates information in various forms, checks for consistency, and “organizes” memories. This is when the hippocampus reviews the information and decides whether it is necessary or not. The bilateral hippocampus is the key brain region of the DMN. Without the rest time, the hippocampus is not given the opportunity or time to sort out and select information, and a lot of information can be discarded because the hippocampus has no time to process. At rest, the hippocampus also helps the brain repair neurons and remove toxins and trash from the brain. As long as the information is not input into the brain, that is, when taking a rest, the hippocampus starts processing information, which can increase memory and generate inspiration. At rest, the prefrontal lobe shuts down, but the hippocampus works all the time, transferring knowledge and experiences from previous short-term memory to long-term memory and consolidating them. Through the function of the hippocampus, the information that needs to be remembered is engraved into the cerebral cortex and transformed into long-term memory. Learning is not just hard work with certain effective methods, rest also plays an essential role in it. Without rest, the brain cannot enter the default mode, and the learning effect is greatly reduced.

### 5.4. Recurrent Neural Replay Occurring in the Brain during Rest

Neural replay consolidates memory by activating activity in relevant brain regions at rest, and replay may be performed in the forward or reverse order in which learning occurs [68,69]. It has been shown that neural replay events occur in the medial temporal cortex and sensorimotor cortex in the DMN during rest [6,69]. The medial temporal cortex includes the hippocampus and entorhinal cortex, which help encode memory for abstract information. The sensorimotor cortex includes brain regions that process sensory information, plan, and perform movements. Neural replay events in the hippocampus and sensorimotor cortex can help consolidate the memories of complex skills, integrate memories associated with abstract knowledge, and motor task planning and execution [68]. Neural replay is more frequent during rest, and the frequency of these neural replay events during rest correlates with the degree of improvement in task performance. Rapid and recurring neural replay events at rest after learning can strengthen the coordination between relevant brain regions, thereby consolidating memory [70].

The above four kinds of neural activities in the brain at rest fully testify that rest promotes learning. The DMN plays an important role in these neural activities occurring at rest. During rest, the brain waves in the DMN regions switch and change rhythmically, and the beta wave rhythm controls behavior and improves attention, memory, and problem-solving ability. The initiation of the “subconscious” divergent thinking mode is actually a switch to the default mode of the brain, allowing neurons to connect freely. The hippocampus is the core region of the DMN, which plays a key role in memory transformation and consolidation. Neural replay is the neural mechanism of the specific expression of the hippocampal force. The DMN is essential to learning. Rest can promote learning and truly achieve the combination of work and rest.

## 6. Discussion and Conclusions

The neural mechanism by which rest promotes learning is complex, and it is also challenging for neuroscience. Based on the discovery, function, and characteristics of DMN, this paper explores the close relationship between the DMN and learning activities at rest, and we discuss several neural activities related to the DMN at rest to support the idea that rest promotes learning. Future exploration of the mechanism of the DMN, the function of different DMN regions, and the collaboration with other brain networks will further improve the comprehensive understanding of the DMN.

How to make better use of the DMN to promote learning and how to help individuals switch to the DMN are all worthy of study. For example, the way to help individuals make plans for learning and rest, and to make plans that are more consistent with the activity law of the DMN, can help improve learning efficiency. To explore these areas, the relationship between the DMN and individual learning and development needs further study. The conclusion that the DMN develops in an inverted “U” shape with age is just a general trend of the development, and the time node of the development change is still unclear. The approximate age at which the DMN develops into the normal adult pattern and becomes aging is still unknown, and the normal level of the default mode state in different ages is still difficult to reveal. The relationship between the DMN and individual psychological development also needs to be further studied, and the interaction mechanism between individual self-awareness development and the DMN is not clear.

There has been some debate about the function and assumptions of the DMN. Only by settling the assumption argument can we better understand its role in learning. Although the function of the DMN has been revealed from the macro level, the exact function has not been completely defined so far. There are two functional theoretical hypotheses of DMN: internal mental processing hypothesis and vigilance hypothesis [2]. The internal mental processing hypothesis points out that the DMN’s function is to process self-reference, episodic memory, mental time travel, and theory of mind, while the vigilance hypothesis holds that the DMN’s function is to maintain extensive attention to the external environment. In fact, the two theories are not mutually exclusive, and cognitive neuroscientists need to get better at bringing them together. The relationship between the DMN and other networks such as visual network, auditory network, and language network remains at the simple switching level, and the specific relationship will be the focus of future research.

The application of the DMN in non-disease fields should be strengthened, especially the application of the DMN in learning field. However, a large number of studies have focused on revealing the relationship between the DMN and mental diseases, such as depression, post-traumatic stress disorder, schizophrenia, ASD, ADHD, and other diseases that are related to the abnormal activity of the DMN. Less attention has been paid to the relationship between other non-disease behaviors and the DMN, and there is a lack of specific research on the use of the DMN. This paper attempts to explore the role of rest in learning from the perspective of the DMN. More research is needed to reveal how to efficiently rest and switch to default mode.

## Data Availability

Not applicable.
